# Microbial responses to soil cooling might explain increases in microbial biomass in winter

**DOI:** 10.1007/s10533-023-01050-x

**Published:** 2023-05-25

**Authors:** Jörg Schnecker, Felix Spiegel, Yue Li, Andreas Richter, Taru Sandén, Heide Spiegel, Sophie Zechmeister-Boltenstern, Lucia Fuchslueger

**Affiliations:** 1 Centre for Microbiology and Environmental Systems Science, University of Vienna, Vienna, Austria; 2 South China Botanical Garden, Chinese Academy of Sciences, Guangzhou, China; 3 Department for Soil Health and Plant Nutrition, Austrian Agency for Health and Food Safety (AGES), Vienna, Austria; 4 Institute of Soil Research, University of Natural Resources and Life Sciences, Vienna, Austria

**Keywords:** Microbial physiology, Carbon cycle, Winter dynamics, Microbial growth, Respiration

## Abstract

In temperate, boreal and arctic soil systems, microbial biomass often increases during winter and decreases again in spring. This build-up and release of microbial carbon could potentially lead to a stabilization of soil carbon during winter times. Whether this increase is caused by changes in microbial physiology, in community composition, or by changed substrate allocation within microbes or communities is unclear. In a laboratory incubation study, we looked into microbial respiration and growth, as well as microbial glucose uptake and carbon resource partitioning in response to cooling. Soils taken from a temperate beech forest and temperate cropland system in October 2020, were cooled down from field temperature of 11 °C to 1 °C. We determined microbial growth using ^18^O-incorporation into DNA after the first two days of cooling and after an acclimation phase of 9 days; in addition, we traced ^13^C-labelled glucose into microbial biomass, CO_2_ respired from the soil, and into microbial phospholipid fatty acids (PLFAs). Our results show that the studied soil microbial communities responded strongly to soil cooling. The ^18^O data showed that growth and cell division were reduced when soils were cooled from 11 to 1 °C. Total respiration was also reduced but glucose uptake and glucose-derived respiration were unchanged. We found that microbes increased the investment of glucose-derived carbon in unsaturated phospholipid fatty acids at colder temperatures. Since unsaturated fatty acids retain fluidity at lower temperatures compared to saturated fatty acids, this could be interpreted as a precaution to reduced temperatures. Together with the maintained glucose uptake and reduced cell division, our findings show an immediate response of soil microorganisms to soil cooling, potentially to prepare for freezing events. The discrepancy between C uptake and cell division could explain previously observed high microbial biomass carbon in temperate soils in winter.

**Supplementary Information:**

The online version contains supplementary material available at 10.1007/s10533-023-01050-x.

## Introduction

In ecosystems with pronounced seasonality, i.e. temperate, boreal, and arctic systems, warm summers are usually considered to be a peak time for soil microbial activity, whereas winter months are described as a time of rest for soil microorganisms (Žifčáková et al. 2017). However, several studies have showen the opposite pattern: an increase in microbial biomass during winter followed by a decrease in spring (Brooks et al. 1998; Jefferies et al. 2010; Isobe et al. 2018). During winter, microbial biomass can act as a buffer for nitrogen, which then is released in spring when plants could take it up again (Schmidt and Lipson 2004; Kaiser et al. 2011; Isobe et al. 2018; Marañón-Jiménez et al. 2019). A similar buffer effect could also occur for C, which gets taken up, transformed and preserved by microorganisms in winter. If microbial C is released in form of microbial necromass in spring, it could be stabilized on soil minerals or in aggregates (Kallenbach et al. 2016; Sokol et al. 2022) and could be an important precursor of soil organic matter (Miltner et al. 2011). The increase in microbial biomass in winter and decrease in spring might thus constitute a ‘prime time’ for C stabilization in soil that is threatened by predicted increased winter temperatures (IPCC 2022).

The mechanisms of microbial biomass increase during winter are not fully understood. One reason could be that a higher proportion of C taken up by the microorganisms is allocated to microbial growth compared to being mineralized and released as CO_2_. This increase in microbial carbon use efficiency (CUE) at lower temperatures (Frey et al. 2013) can derive from a higher temperature sensitivity of respiration than of growth (Pietikäinen et al. 2005; Cruz-Paredes et al. 2021), allowing microorganisms to increase microbial biomass. Alternatively, microbial biomass could increase because fewer microorganisms fall victim to predation. Predatory species in soil often have higher temperature limits and optima than their prey (Geisen 2021). At low temperatures, predators could thus already be inactive while their prey microorganisms could still build up their biomass. A third possible mechanism is that soil microorganisms increase their cell contents in winter, either as storage (Manzoni et al. 2021; Mason-Jones et al. 2022) in preparation for substrate shortages, or as osmolytes or cryoprotectants (Drotz et al. 2010). As soils gradually freeze, nutrient and salt concentrations in the remaining liquid phase increase and put osmotic stress on microbial cells in the soil (Rivkina et al. 2000; Jefferies et al. 2010). If temperatures drop further, microbes themselves could freeze and lyse in the process (Drotz et al. 2010; Tribelli and López 2018). To counteract this stress as well as the reversed effect during thawing, microorganisms have been shown to produce osmolytes at temperatures below freezing (Drotz et al. 2010; Panikov et al. 2006).

Studies investigating cooling effects on microbial processes and activities have been carried out at temperatures below freezing (Panikov et al. 2006; Drotz et al. 2010), or after freeze-thaw cycles (Sorensen et al. 2018). We here focus on a temperature of 1 °C, just above freezing, and ask if we can detect cooling responses in microbial respiration, growth, C uptake, and allocation. Soil microbial communities either respond immediately to changes in their environment or can adapt to prolonged periods of stress through physiological adjustments (Schimel et al. 2007; Jansson and Hofmockel 2020). We therefore further investigated whether cooling responses happen after the first two days of cooling or if responses changed after an acclimation period of nine days.

In a short-term lab incubation study, we cooled down soils from a temperate cropland and a temperate beech forest from 11 °C field temperature to 1 °C and measured DNA-based microbial growth, microbial C uptake and allocation into respiration and phospholipid fatty acids. We cooled down soils after soil sampling from the field sites to determine the immediate responses to cooling, and we let the soils acclimate to low temperatures for nine days before we determined the same parameters again. We hypothesized that 10 degrees cooling would decrease all microbial processes including C uptake, respiration, and growth. We further hypothesized that after nine days of cooling microbial communities would have acclimated to colder temperatures on the one hand indicated by a shift in microbial community composition determined by PLFAs, but also by changing activity pattern favoring C uptake and storage over allocation to growth and respiration.

## Materials and methods

### Study sites

Soil samples were collected from an agricultural field site and a deciduous forest. Agricultural soils were sampled at a long-term agricultural field experiment near Wieselburg, in Alpenvorland, Austria (48°12′N 15°15′E), established in 1986. Mean annual temperature (MAT) at the site is 8.5 °C and mean annual precipitation (MAP) is around 840 mm. The soil is classified as gleyic Luvisol (Spiegel et al. 2018) and has a silt loam texture (10% sand, 73% silt, and 17% clay). Soil pH was 6.1 (Canarini et al. 2020). At the time of sampling in October 2020, the field was bare after winter barley had been harvested in the preceding summer. The forest study site is located at the experimental forest Rosalia, Austria (47°42′N, 16°17′E) and is dominated by European beech (Fagus sylvatica L.). The soil at the site is a gleyic Cambisol (Leitner et al. 2016). Texture is a sandy loam (55% sand, 38% silt, and 7% clay), soil pH is 4.9 (Canarini et al. 2020). Soils were sampled from both sites in October 2020 with a soil corer with a diameter of 2 cm from 0 to 5 cm depth. Soil water content at the time of sampling was on average 20.4% of dry soil or 53% of water holding capacity (WHC) for agricultural soils and 34.5% of dry soil (46% WHC) for forest soils. At both sites, we sampled four replicate plots. We took 10 soil cores from each plot and combined them to one composite sample. This resulted in four replicates for each field site. At the agricultural site, the four sampled plots were 7.5 m wide and 28 m long and at least 5 m apart from the next plot. At the forest site, the 3 m by 3 m plots were at least 10 m apart from each other. All composite soil samples were homogenized by sieving through a 2 mm mesh in the field and transported to the lab in plastic bags. There the soil samples were put in an incubator set to the field temperature of 11 °C until the next day when the incubation experiment started. Also, from each of the 8 samples, about 5 g of field moist soil were weighed into aluminum dishes and put in a drying oven at 105 °C overnight. On the next morning the aluminum dishes were weighed again to estimate water content of the soils which was necessary for the water and substrate additions later that day. More precise determinations of soil water content were conducted later on.

### Experimental setup

We set up a laboratory incubation experiment to determine the response of soil microorganisms to immediate cooling and the short-term acclimation of microorganisms to cold temperatures. We used ^18^O and ^13^C tracing approaches to determine microbial processes. The isotope assays were started on the day after soil sampling which marks timepoint zero of the cooling experiment. Isotope assays were performed in two sets: One set was incubated at 11 °C and one at 1 °C for 42 h to determine the immediate response to cooling. Additionally, the remaining soil was split in two and one part was incubated at 11 °C and the other at 1 °C. After 144 h, we again started the isotope assays for the respective temperatures, which ended at 186 h of the total runtime of the cooling experiment to determine short-term acclimation of microorganisms to cold temperatures.

#### Immediate cooling response

In the morning after soil-sampling, we weighed the following amounts from each of the 8 soil samples into vials and containers for specific methods to determine microbial and soil properties:

Two sets of four 1.2 mL plastic cryovials were filled with 400 mg soil each to determine microbial growth using ^18^O incorporation in DNA and CUE (^18^O-tracing). One set was designated for growth and CUE measurements at 11 °C, the other set at 1 °C. Details for incubations and further processing are stated below (Sect. "Microbial growth, and carbon use efficiency (18O tracing)").

Two sets of two 50 mL glass headspace vials were filled with 10 g of soil each to traced ^13^C from amended glucose into extractable organic C (EOC), microbial biomass C (MBC), and respired C in CO_2_ as well as in phospholipid fatty acids (PLFA). One set was designated for ^13^C-tracing at 11 °C, the other set at 1 °C. Details for incubations and further processing are stated below (Sect. "Microbial C uptake and allocation (13C tracing)").

An aluminum dish was filled with 5 g of soil and put in a drying oven at 105 °C for 24 h to determine soil water content. Afterwards, to determine soil organic C content, dried samples were finely ground in a ball mill, before aliquots were packed in tin capsules and measured on an elemental analyzer-isotope ratio mass spectrometry (EA-IRMS; CE Instrument EA 1110 elemental analyzer, coupled to a Finnigan MAT DeltaPlus IRMS with a Finnigan MAT ConFlo II Interface).

#### Short-term acclimation of microorganisms to cold temperatures

On the day after soil sampling, and after weighing out the above-mentioned aliquots, the soil remaining in the plastic bags was roughly divided in two aliquots (about 50 g) and enclosed in air filled plastic bags. One aliquot of each initial soil sample was placed in an incubator set to 11 °C and the other aliquot was placed in an incubator set to 1 °C.

After 144 h of the cooling experiment, we used the soils in the plastic bags. We weighed in soil aliquots from each of the 16 soil/temperature combinations in a similar manner as described above, with the exception that for the ^18^O and ^13^C approaches only one set of samples was prepared for each soil/temperature combination, which was then returned to its previous temperature.

### Microbial growth, and carbon use efficiency (^18^O tracing)

Microbial growth and carbon use efficiency (CUE) were determined following Spohn et al. (2016) and Zheng et al. (2019) with slight modifications. The pre-weighed sets of samples designated for ^18^O tracing were taken out of the respective incubators to add substrate and 18O tracers and to start the isotope tracing assay. This was done as fast as possible and the samples were handled at room temperature for less than 10 min. The pre-weighed sets were opened and the plastic vials were inserted into 27 mL headspace vials, which were sealed with a rubber septum. This was done to inhibit exchange of the later amended ^18^O-water with the surrounding air and to allow measurement of CO_2_ accumulation over time to determine respiration rates. Using a syringe and needle the following combinations of water (at room temperature) and glucose were added: natural abundance water, ^18^O-labelled water, natural abundance water plus glucose, and ^18^O water plus glucose. Water was added to achieve 60% water holding capacity and 20 atm% ^18^O in the final soil water, when ^18^O-water was added. We aimed to add C in the form of Glucose to amount to 20% of MBC. Both WHC and MBC had been determined previously for the sites (Schnecker et al. 2022). We added 10 µg Glucose-C per g dry soil to agricultural soils and 50 µg C per g dry soil to forest soils, which amounts to 19% and 17% of the respective C in microbial biomass in this incubation experiment. The same C additions were chosen for the ^13^C tracing experiment.

Once the water was added, we measured the CO_2_ concentration in the headspace vials by taking gas samples from the sealed headspace vial and measured it directly with an infrared gas analyzer (EGM4, PP systems). The removed air from the headspace vial was replaced with the same amount of synthetic air with known CO_2_ concentration. Afterwards, the sets were returned to the incubators. At timepoint zero, one set was incubated at 11 °C and the other at 1 °C. After 144 h of the cooling experiment, the sets were returned to the incubators where they had been in before i.e., set to 11 °C or 1 °C. Samples remained in the incubator for 42 h (until hour 42 and hour 186 of the cooling experiment respectively) after which another CO_2_ sample was taken and measured. Microbial respiration was then calculated as the difference in CO_2_ concentrations between those two time points and accounting for the replaced air, divided by the incubation time.

After the CO_2_ measurement, the headspace vials were opened, the plastic cryovials were taken out, closed, shock-frozen in liquid N and stored for later extraction of DNA. Microbial growth was determined based on the incorporation of ^18^O from soil water into DNA. DNA was extracted using a DNA extraction kit (FastDNA™ SPIN Kit for Soil, MP Biomedicals) following the manufacturer’s instructions. The DNA concentration of each extract was determined fluorometrically by a Picogreen assay using a kit (Quant-iT™ PicoGreen® dsDNA Reagent, Life Technologies). Subsequently, total oxygen content and ^18^O enrichment of the purified DNA fractions were measured using a thermochemical elemental analyzer (TC/EA, Thermo Fisher) coupled via a Conflo III open split system to an isotope ratio mass spectrometer (Delta V Advantage, Thermo Fisher).

The amount of DNA produced was calculated using the following formula:

$${\text{DNA}}_{{{\text{produced}}}} = {\text{O}}_{{{\text{DNA extr}}}} *\frac{{^{{18}} {\text{O at\% }}_{{{\text{DNA L}}}} - ^{{18}} {\text{O at\% }}_{{{\text{DNA}}{\kern 1pt} {\text{n}}{\text{.a}}{\text{.}}}} }}{{^{{18}} {\text{O at\% }}_{{{\text{soil water}}}} }}*\frac{{100}}{{31.21}}$$

where *O*_*DNA extr*_ is the total amount of oxygen in the DNA extract, ^*18*^*O at%*_*DNA L*_ and ^*18*^*O at%*_*DNA n.*a_. are the ^18^O enrichment in the labeled and unlabeled DNA extracts respectively, and ^*18*^*O at%*_*soilwater*_ is the ^18^O enrichment of the soil water. The fraction at the end of the formula accounts for the average oxygen content of DNA (31.21%, Zheng et al. 2019; Canarini et al. 2020). To calculate microbial biomass C produced (C_Growth_) during the incubation, *DNA*_*produced*_ was divided by the total amount of DNA in the sample and multiplied by MBC values. Microbial respiration (C_Respiration_) was calculated from the respiration measurements described above.

Microbial CUE was calculated using the following equation (Manzoni et al.,^ 2012^):

$${\text{CUE}} = \frac{{{\text{C}}_{{{\text{Growth}}}} }}{{{\text{C}}_{{{\text{Growth}}}} {\text{ + C}}_{{{\text{Respiration}}}} }}$$

### Microbial C uptake and allocation (^13^C tracing)

To trace ^13^C into extractable organic C, MBC, CO_2,_ and PLFAs, the sets designated for ^13^C-tracing were amended either with 10 atm% ^13^ C-labelled glucose solution or natural abundance water at timepoint zero and 144 h of the cooling experiment. To add the solutions and start the ^13^C assay, the samples were briefly removed from the incubators for less than 10 min. The amount and concentration of glucose and water was the same as for the above described ^18^O-tracing. After the addition of ^13^C-glucose, and water which was added to a control set, the 50 mL headspace vials were closed with a rubber septum. To determine respiration and to trace ^13^C into CO_2_, 15 mL of the headspace air were sampled with a syringe and needle and transferred to pre-evacuated gas vials. The air was replaced with 15 mL synthetic air.

On timepoint 0, one set of samples was then incubated at 11 °C while the other set was put in an incubator set to 1 °C. After 42 and 186 hours gas samples were taken. Gas samples were analyzed for their CO_2_ concentrations and δ^13^C signatures by a headspace gas sampler (GasBench II, Thermo Fisher, Bremen, Germany) coupled to an isotope ratio mass spectrometer (Delta V Advantage, Thermo Fisher, Bremen, Germany). Microbial respiration was then calculated as the difference in CO_2_ between the two sampling time points, accounting for the replaced air, and divided by the incubation time.

After the gas sampling, the headspace vials were opened. From each vial 2 g of soil were weighed in 20 mL plastic scintillation vials and immediately amended with 15 mL 0.5 M K_2_SO_4_. The vials were then closed and shaken horizontally on a shaker for 30 min and afterwards filtered through ash-less paper filters. Also 2 g of soil from the 50 mL headspace vials were weighed in 20 mL glass scintillation vials. The glass scintillation vials were used or chloroform fumigation and received a 1.5 mL glass vial containing 150 µL CHCl3 and were closed with a PTFE-coated septum. The glass vials were then returned to the respective incubators for 48 h. Afterwards, the 1.5 mL glass vial containing the chloroform was removed and the samples were left open in a fume hood to aerate for 30 min. Afterwards, the samples were extracted as described above with 15 mL 0.5 M K_2_SO_4_. Chloroform fumigation was also performed at the same temperature at which the respective sets had been incubated. Both extracts, from fumigated and non-fumigated soils, were measured on a TOC/TN analyzer (TOC-L CPH/CPN, Shimadzu) to determine EOC and MBC (Brookes et al. 1985) and by direct injection on an IC system (DX 3000, Dionex Corporation, Sunnyvale, CA, USA) without column and connected through a Finnigan LC IsoLink Interface (Thermo Fisher Scientific, Waltham, MA, USA) to a Finnigan Delta V Advantage Mass Spectrometer (Thermo Fisher, Bremen, Germany) to determine ^13^C incorporation into microbial biomass. MBC was calculated as the difference in C between fumigated samples and fresh soil samples. Measured MBC values were divided by 0.45 (Wu et al. 1990) to account for extraction efficiency. Carbon substrate incorporation into microbial biomass was calculated as the difference between ^13^C in EOC of chloroform-fumigated and non-fumigated samples.

From the headspace vials containing agricultural soils 2 g, and from the headspace vials containing forest soils 1 g, were frozen at − 20 °C and freeze-dried for later extraction of PLFAs.

### Phospholipid fatty acids

Phospholipid fatty acids (PLFAs) of major microbial groups were estimated by extracting PLFAs from freeze-dried soil samples using a modified high throughput method based on Buyer and Sasser (2012). Total lipids were extracted from soils using a chloroform/methanol/citric acid buffer mixture and fractionated by solid-phase extraction on silica columns. The PLFA fraction was collected by eluting columns with a 5:5:1 chloroform:methanol:water mixture. After an internal standard (19:0) was added, PLFAs were converted to fatty acid methyl esters (FAMEs) by transesterification. Samples were analyzed for quantification and ^13^C incorporation using a Trace GC Ultra connected by a GC-IsoLink to a Delta V Advantage Mass Spectrometer (all Thermo Fisher Scientific). FAMEs were identified using mixtures of bacterial and fungal FAMEs (Bacterial Acid Methyl Ester CP Mixture; Matreya LLC, State College, PA, USA) and 37 Comp. FAME Mix (Supelco, Bellefonte, PA, USA) and quantified against an internal standard (19:0). We used the markers 18:1ω9cis and 18:2ω6,9 to estimate fungal biomass (Frostegård et al. 2011). The sum of a15:0, i16:0, 16:1ω7, 10Me16:0, a17:0, cy17:0, and 10Me18:0 a used as Gram‐positive bacterial. The markers 16:1ω7, cy17:0, and cy19:0 were used as bacterial markers (Quideau et al. 2016). The remaining peaks including the PLFA general markers 15:0, 16:1ω5, 16:1ω9, 18:1ω9trans, 19:1ω9, 16:0, and 17:0 are not exclusive bacterial nor fungi markers and were assigned to the general PLFA marker group (Quideau et al. 2016). We also grouped markers into saturated and unsaturated PLFAs. We calculated relative abundance of PLFAs by dividing the respective amount of C in a biomarker by the sum of all biomarkers. ^13^C label allocation to bacteria, fungi, and unsaturated PLFAs was calculated by dividing the sum of ^13^ C in the respective PLFAs of this group by the total amount of ^13^C in all determined PLFAs.

### Response ratio to cooling (RR)

Response ratios to cooling for respiration and growth, CUE, label respiration, label incorporation in microbial biomass and in PLFA was calculated for both, immediate response to cooling (timepoint zero until 42 h) and short-term acclimation (144–186 h) and each site as well as treatment (glucose addition) individually using the following formula:

$${\text{RR}} = \frac{{{\text{R}}_{{11}} }}{{{\text{R}}_{1} }}$$

where R_1_ and R_11_ represent respiration and growth, CUE, label respiration, label incorporation in microbial biomass and in PLFA at 1 ℃ and 11 ℃ respectively.

### Statistics

Measurements for both sites and all treatments (combinations of temperature, glucose or water additions, day of incubation) were performed for all 4 plot replicates at each site.

All statistical analyses were performed in R 4.1.1 (R Development Core Team 2013). To test for differences in all measured factors between sites and effects of temperature, glucose addition, or duration of cooling, we used Fit Linear Model Using Generalized Least Squares (R function ‘gls’) and Linear Mixed-Effects Models (‘lme’), which are both contained in the R package ‘nlme’ (Pinheiro et al. 2021). To account for non-normal distributed residuals, we used log transformations where necessary. If residuals of the models were non-homoscedastic, we introduced weights in the respective functions. We also introduced field plots as random effects. Different models including weights and random effects were set up and compared with the ANOVS (‘anova’). If models were statistically different, we chose the model with the lowest Akaike information criterion (AIC).

To visualize PLFA patterns and to determine differences in the composition of PLFA patterns, we created nmds (Nonmetric Multidimensional Scaling) matrixes (R function ‘metaMDS’) of the relative abundances of all measured PLFAs to create. We used ‘envfit’ to determine the PLFA markers, which significantly determined the distribution in the nmds plots, and tested the effects of incubation temperature, glucose addition or sampling time point on PLFA patterns using the Permutational Multivariate Analysis of Variance Using Distance Matrices (‘adonis’-function). ‘nmds’, ‘envfit’, and ‘adonis’ are part of the ‘vegan’-package (Oksanen et al. 2013). Statistical tests were assumed to be significant at p < 0.05.

## Results

All isotope-tracing approaches, that were started on timepoint 0 integrate over the first 42 h and thus represent the immediate response to cooling and are labelled with “42 h” in the graphs below and referred to as ”42 h” in this results section. All results for the determination of short-term acclimation, which cover 144–186 h of the cooling experiment will be labelled and referred to as “186 h”. Microbial biomass measurements are considered to correspond to the time point when the fumigation was started which was at the end of the ^13^C-tracing approaches, i.e. after 42 h of cooling and after 186 h of cooling.

### Differences between sites

After 42 h of the experiment at 11 °C, SOC, MBC, and total PLFAs were 5.1, 6.5, and 13.6 times higher, respectively, in forest soils compared to agricultural soils (Table 1). Similarly, microbial respiration and growth were 8.1 and 6.4 times higher in forest soils, while ^13^C substrate-derived respiration was 4.4 times higher and label incorporation in microbial biomass was 3.9 times higher in forest soils compared to agricultural soils. CUE was not different between sites (Table 1).

**Table 1:** Differences between soil from agricultural site and forest site. Given are the mean values after 42 h of the experiment at 11 °C plus the respective standard error

	Agricultural	Forest	F-value	p-value
SOC (mg C g^−1^ dry soil)	8.864 ± 0.213	45.641 ± 2.365	**239.8**	**< 0.001**
MBC (µg C g^−1^ dry soil)	45.15 ± 3.894	294.3 ± 27.64	**214.1**	**< 0.001**
total PLFA content (nmol C g^−1^ dry soil)	145.3 ± 44.77	1975 ± 215.3	**34.45**	**0.001**
microbial respiration (ng C h^−1^ g^−1^ dry soil)	119.9 ± 2.482	975.2 ± 68.00	**721.2**	**< 0.001**
microbial growth (ng C h^−1^ g^−1^ dry soil)	87.98 ± 8.884	569.9 ± 86.7	**98.46**	**< 0.001**
CUE	0.420 ± 0.027	0.364 ± 0.026	2.261	0.183
label derived respiration (ng C h^−1^ g^−1^ dry soil)	23.11 ± 0.077	101.1 ± 111.6	**176.4**	**< 0.001**
label incorporation in microbial biomass (ng C h^−1^ g^−1^ dry soil)	58.29 ± 1.471	229.4 ± 33.38	**100.8**	**< 0.001**

Statistically significant effects are indicated by bold F-values and p-values

### Fast effects versus acclimation responses to cooling of microbial respiration, growth, biomass, and carbon use efficiency

**Fig. 1:**
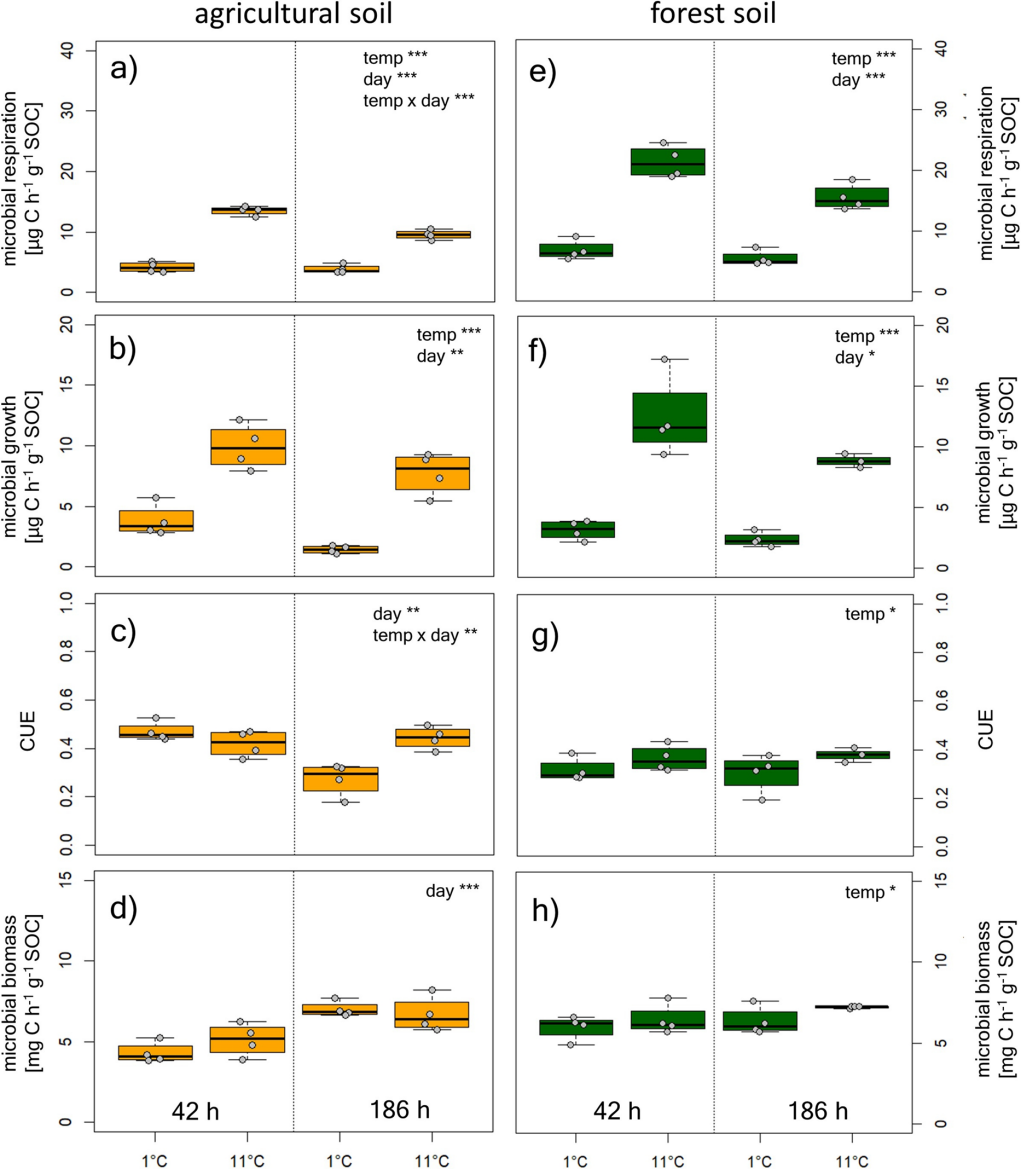
Microbial Respiration, growth carbon use efficiency and microbial biomass C of agricultural soil (**a**–**d**) and forest soil (**e–h**) at different temperatures (1 and 11 °C) during immediate cooling (42 h) and after acclimation to the temperatures (186 h). Statistics accompanying this figure can be found in Table S1

Initial cooling of soils from 11 °C to 1 °C significantly reduced microbial respiration (Fig. 1a, e) growth (Fig. 1b, f) and carbon use efficiency (Fig. 1c, g) in both agricultural and forest soils. Microbial respiration and growth were on average reduced by 57% and 71% respectively in agricultural and by 64% and 74% in forest soils as a consequence of cooling (calculated per g dry soil, Table S4). MBC was lower at the beginning of the incubation in agricultural soils and increased over time. In forest soils, MBC was consistently lower at 1 °C than at 11 °C (Fig. 1d, h).

The calculated RR which are the factor by which the measured rates and parameters were lower at 1 °C compared to at 11 °C were not significantly different between sites. RR for respiration ranged from 2.61 ± 0.19 to 3.30 ± 0.25 and for growth from 2.74 ± 0.35 to 5.92 ± 0.94 (Table 2). CUE was significantly reduced at lower temperatures (Fig. 1), and calculated RR were lower than those for respiration and growth ranging from 0.89 ± 0.04 to 1.72 ± 0.25 (Table 2).

**Table 2:** Response ratios to cooling for the measured parameters Given are the mean values calculated for the respective day of the incubation plus standard error

			Microbial respiration	Microbial growth	CUE	Label respiration	Label in mic. biomass	Label in PLFA
Agricultural		42 h	3.30 ± 0.25	2.74 ± 0.35	0.89 ± 0.04	1.41 ± 0.05	0.78 ± 0.05	1.51 ± 0.20
		186 h	2.61 ± 0.19	5.92 ± 0.94	1.72 ± 0.25	1.36 ± 0.02	0.97 ± 0.10	1.42 ± 0.20
		F-value	**27.21**	**7.526**	**7.784**	0.651	2.268	1.636
		p-value	**0.014**	**0.033**	**0.032**	0.454	0.183	0.291
Forest		42 h	3.28 ± 0.04	4.26 ± 0.59	1.18 ± 0.11	1.20 ± 0.05	1.04 ± 0.14	1.6 ± 0.13
		186 h	2.79 ± 0.12	3.43 ± 0.16	1.11 ± 0.05	1.34 ± 0.02	0.98 ± 0.14	1.19 ± 0.28
		F-value	**11.528**	1.139	0.160	4.106	0.070	1.508
		p-value	**0.015**	0.292	0.706	0.089	0.801	0.274

Statistically significant differences in the response ratios between 42 and 186 h are indicated by bold F-values and p-values

RR of respiration were significantly lower after 186 h compared to after 42 h of cooling at both sites. While no differences in RR of growth and CUE between 42 and 186 h of cooling were found in forest soils, both RR increased in agricultural soils over time (Table 2).

### Cooling effects on glucose respiration and incorporation into microbial biomass

**Fig. 2:**
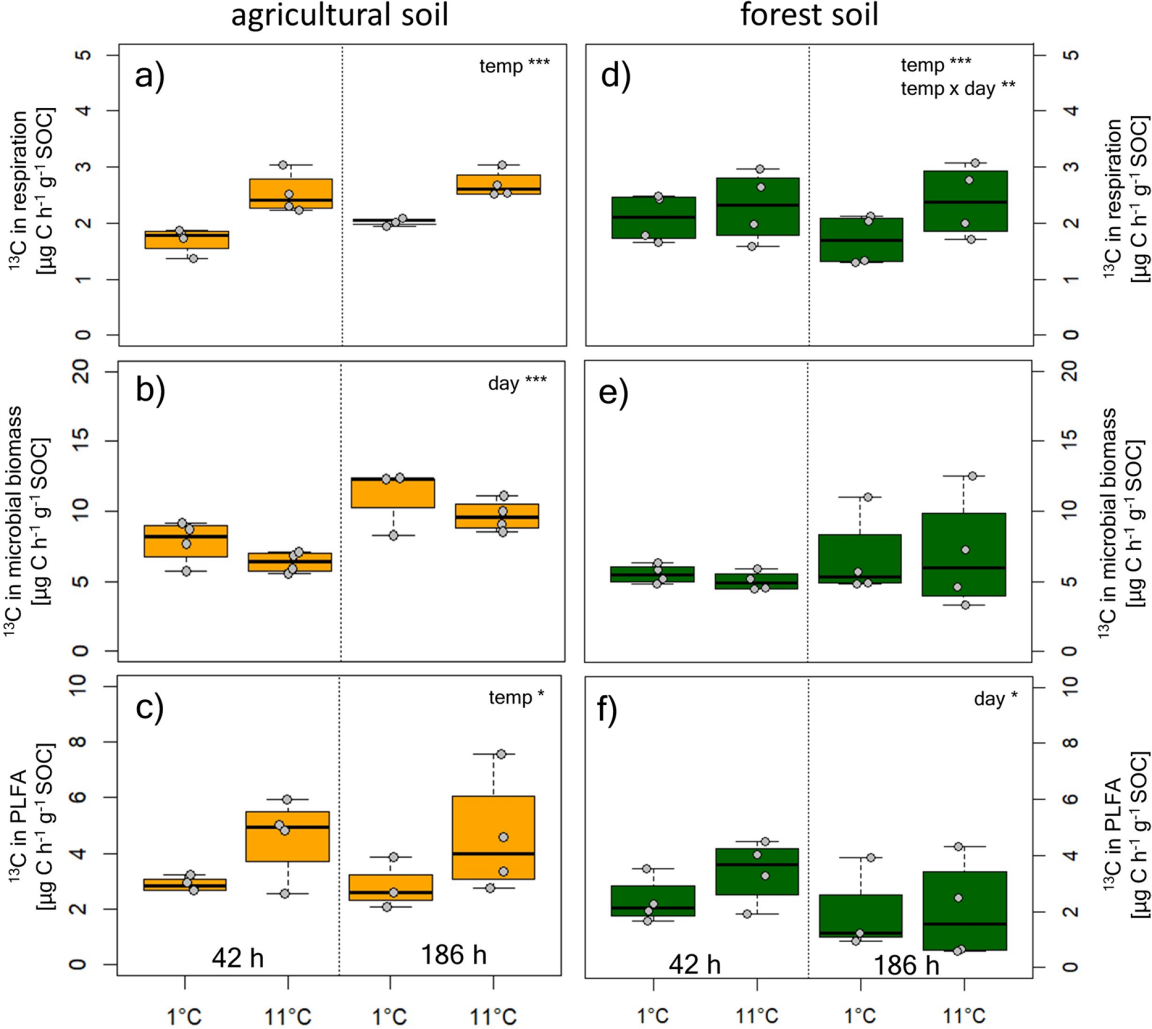
^13^C-Glucose-derived respiration and recovery in in the MBC pool, as well as in total PLFAs (42 h after addition) in agricultural soils (**a**–**c**) and forest soils (**d–f**) at different temperatures (1 and 11 °C) during immediate cooling (42 h) and after acclimation to the temperatures (186 h). Statistics accompanying this figure can be found in Tables S1 and S2

We found significant effects of cooling on the use of labile C after 42 and 186 h (Fig. 2). Label-derived respiration at both sites was significantly lower at 1 °C than at 11 °C. Label incorporation in microbial biomass was not affected by temperature and was as high at 1 °C as at 11 °C in agricultural soils and forest soils, but increased from 42 to 186 h in the agricultural soil (Fig. 2b, e). Label incorporation in PLFAs was elevated by temperature in agricultural soils and decreased from 42 to 186 h in forest soil. In general label uptake and respiration showed lower RR than total respiration and microbial growth indicating less sensitivity to cooling (Table 2).

### Effects of cooling on microbial community composition and glucose allocation to PLFAs

**Fig. 3:**
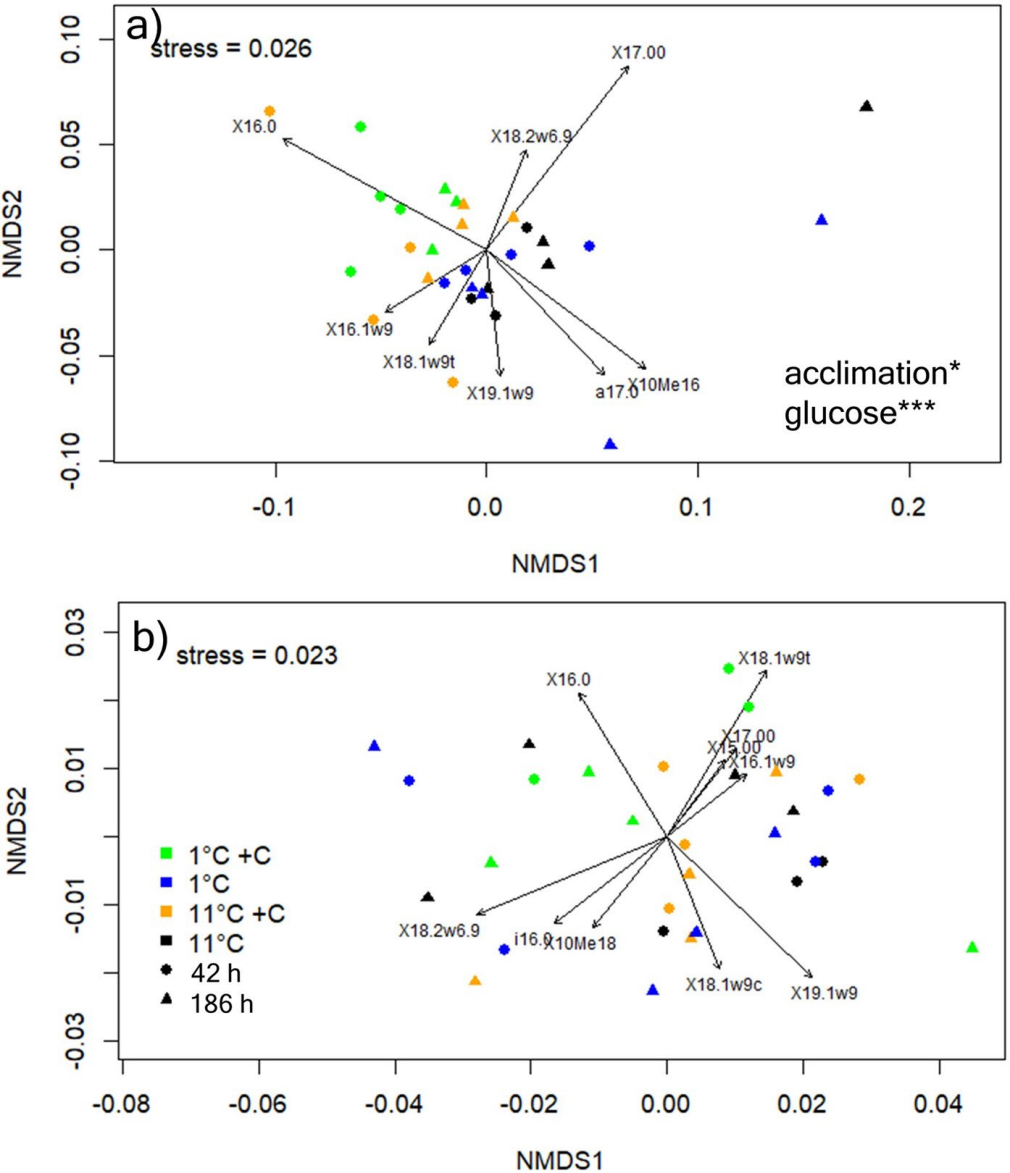
NMDS plots of PLFAs in the agricultural soil (**a**) and forest soil (**b**). Shown PLFA markers significantly shaped the shown distribution of samples in the NMDS. Samples incubated at 1 °C that received glucose (1 °C + C) are shown in green. Samples incubated at 1 °C that received only water (1 °C) are shown in blue. Samples incubated at 11 °C that received glucose (11 °C + C) are shown in orange. Samples incubated at 11 °C that received only water (11 °C) are shown in black. Samples from the immediate cooling incubation (42 h) are shown as circles, sample from acclimated soils (186 h) are shown as triangles. Agricultural soils showed a significant effect the duration of cooling at 1 °C (acclimation) and of glucose addition. Detailed statistics can be found in Table S3

Microbial community composition, determined by PLFAs and investigated by multidimensional scaling of relative abundances of all individual biomarkers, was strongly different between agricultural and forest soil, but was not after 42 h of cooling (Fig. 3). However, the PLFA patterns at the agricultural site were affected by glucose addition and were different after 42 h compared to after 186 h of cooling suggesting that microbial communities acclimated to colder temperatures over time (Fig. 3a).

**Fig. 4:**
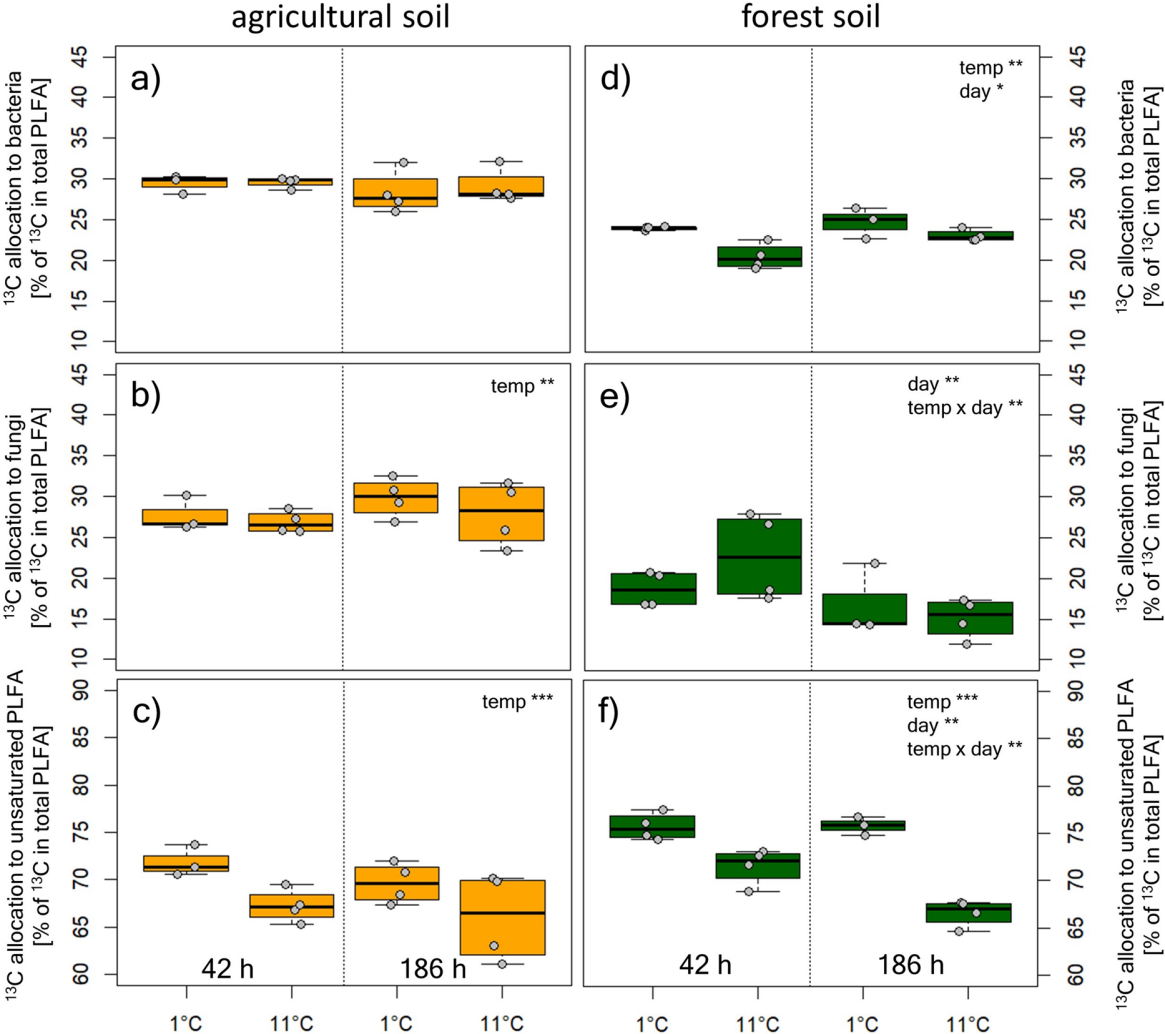
Label allocation to bacterial PLFAs, fungal PLFAs and to unsaturated PLFAs after incubation with ^13^C labelled glucose in agricultural soils (**a–c**) and forest soils (**d–f**) at different temperatures (1 and 11 °C) during immediate cooling (42 h) and after acclimation to the temperatures (186 h). Statistics accompanying this figure can be found in Tables S1 and S2

Moreover, cooling affected the incorporation of ^13^C in total PLFAs and into individual PLFA groups. At both sites, ^13^C incorporation in PLFAs was significantly lower at low temperatures immediately and after seven days of acclimation. In forest soils but not in agricultural soils, more ^13^C was allocated to bacteria at 1 °C than at 11 °C (Fig. 4). In contrast, ^13^C allocation to fungal PLFAs was slightly but significantly higher at low temperatures in agricultural soils, but not in forest soils. In both soils, cooling induced a significantly higher proportion of ^13^C glucose to be allocated to unsaturated fatty acids.

## Discussion

In our study, we investigated microbial responses to cooling to elucidate potential mechanisms behind previously observed increases in microbial biomass in winter. We found that cooling soils from their field temperature at 11 °C to 1 °C reduced microbial respiration and growth. In contrast, cooling had little to no effect on the uptake and respiration rate of amended glucose. The additionally incorporated C might constitute storage compounds or precursors of osmolytes to prepare against changes in osmotic potential. Cooling also led to an increased allocation of C to unsaturated PLFAs. Our results suggest that while increased CUE at lower temperatures (Schnecker et al. 2022) and reduced predation pressure might contribute to increases in microbial biomass in winter, physiological adaptations to counteract cooling and potentially freezing might constitute the main mechanism of previously observed microbial biomass increase during the cold season in temperate soil ecosystems.

### Microbial responses to cooling

In contrast to our hypothesis, microbial processes responded variably to cooling. Microbial respiration and DNA based growth estimates, indicating cell division were strongly decreased. This suggests that winter and cold temperatures, mark a time of microbial rest (Žifčáková et al. 2017). In contrast to other studies, we did not observe a higher temperature sensitivity of respiration than of growth (Pietikäinen et al. 2005; Frey et al. 2013; Cruz-Paredes et al. 2021), which resulted in only minor differences in microbial CUE values at 11 °C and at 1 °C (Fig. 1). Since CUE did not change with temperature in the investigated soils, it is unlikely that a higher allocation of C to growth is responsible for observed increases in MBC in winter. In a previous study at the same sites, we could further show that MBC but not DNA content increased in winter (Schnecker et al. 2022). If higher CUE or less predation would have been responsible for the winter increase in MBC, DNA content should have increased accordingly which makes it unlikely that higher CUE or lower predation pressure are the main mechanisms behind the winter increase in MBC.

Another possible explanation for increases in MBC might be the production of osmolytes inside the microbial cell (Panikov et al. 2006; Drotz et al. 2010). Osmotic stress on microbial cells during freezing is comparable to drought, and thawing of previously frozen soil can have similar consequences as sudden rewetting of dry soils (Jefferies et al. 2010; Schimel 2018). As osmolytes are produced rather fast in response to changes in osmotic potential and cannot be stored or produced in advance, the maintained uptake of C at temperatures just above freezing and concomitant lack of cell division and growth in our study, suggests that taken up C might have been invested in storage compounds (Mason-Jones et al. 2022), either to be used for later growth or for the quick production of osmolytes. During the rather short total incubation time in this study, we could find an increase in MBC from 42 to 186 h of cooling in agricultural soils, although at both incubation temperatures. In the forest soil however, MBC was lower at 1 °C than at 11 °C even after 186 h of cooling. While our short-term incubation experiment could not replicate MBC increases found in the field, over the course of weeks and months at cool temperatures, a continued uptake of available C with concomitant reductions in microbial cell division and respiration, as shown in this experiment, might however lead to previously observed increases in MBC in winter (Schnecker et al. 2022).

Another adaptation to low temperatures in our study was that taken-up glucose was not allocated to specific microbial groups, but was invested at a higher relative proportion in unsaturated PLFAs across the microbial community in cold soils. A change in the composition of microbial cell membranes can be interpreted as a specific microbial community adaptation to cool temperatures (Drotz et al. 2010). The fluidity of the main components of cell membranes, fatty acids, is strongly temperature dependent. Saturated fatty acids change from liquid to solid state at higher temperatures than unsaturated fatty acids (Rilfors and Lindblom 2002). Higher investments into unsaturated fatty acids enhances the flexibility of cell membranes in general, which might be crucial for soil microorganisms at low temperatures and has been repeatedly shown, especially in frozen soils (Panikov et al. 2006; Bore et al. 2019). This might also be related to changes in osmotic potential during freeze-thaw cycles which lead to rather rapid expansion of the cell membrane. A flexible cell membrane is thus of essence, to maintain cell integrity during fluctuations of the osmotic potential. Our data show, that unsaturated fatty acids are preferentially produced as a response to cooling and might even be a preparation of the microbial community for soil freezing.

### Microbial acclimation to cooling

After 186 h of cooling, respiration rates of both soils at 11 °C were lower, than after 42 h while label-derived respiration was not changed. Growth was also slightly lower at 11 °C after 186 h than after 42 h of cooling at the forest site. Freshly amended glucose was however readily taken up in both soils independently of the hour of incubation although the allocation into PLFAs was lower after 186 h compared to 42 h at the forest site. This suggests that increasing incubation time could decrease resource availability for soil microbes (Lipson et al. 2000; Brooks et al. 2005). In addition, we found shifts in the microbial community composition in response to glucose addition and after acclimation in agricultural soil but not in forest soils. While it is surprising that this shift was detected after only 42 h in case of glucose and after only 186 h of incubation at the coarse level that PLFA analyses provide, seasonal changes, and especially changes from summer to winter in the microbial community are well known (Bardgett et al. 1999; Kaiser et al. 2010; Lazzaro et al. 2015; Zhang et al. 2020). These shifts might however again be linked to low substrate availability after seven days of incubation and especially in the agricultural soils that can be considered rather C limited (Soong et al. 2020).

## Conclusion

In this study, we found that microbial respiration and growth (cell division), were strongly reduced during the initial cooling from 11 °C to 1 °C while the uptake and allocation of available glucose remained almost unaffected. We also found that taken up labile C was rather allocated to unsaturated than saturated fatty acids, potentially to maintain membrane flexibility at low temperatures. The maintained uptake of easily available C at low temperatures might have been used for production of storage compounds. Storage compounds may either be used for the rapid production of osmolytes when freezing soil water alters the osmotic potential of the unfrozen soil solution, forcing the microbes to adjust their osmotic potential, or for later use in maintenance or growth.

The fate of C accumulated in the microbial biomass in winter, as well as its potential for stabilization in soils largely depend on the form in which C is present in microbial cells and what the causes for the decrease in MBC in spring are. While these questions remain open, our findings suggest that a major mechanisms of C increase in microbial biomass in winter in soils, is provisioning of carbon for later use (such as the production of osmolytes at temperatures below 0 °C) and to a lesser extent for adaptation of the cell membranes to colder temperatures.

## Supplementary Information

Below is the link to the electronic supplementary material.

Supplementary material 1 (DOCX 410.5 kb)

## Data Availability

The datasets generated during and/or analyzed during the current study will be available in the Phaidra repository upon publication of the manuscript and are for now available from the corresponding author on reasonable request.
