# Supplementary material for: Microbial responses to soil cooling might explain increases in microbial biomass in winter
Source: Biogeochemistry. 2023 May 25;164(3):521–35. doi: 10.1007/s10533-023-01050-x (PMC10354169; doi:10.1007/s10533-023-01050-x)
Supplement: Supplementary file 1 — Supplementary material 1 (DOCX 410.5 kb) [file 10533_2023_1050_MOESM1_ESM.docx]

Table S1 Statistical test results for agricultural soil (n=4). Shown are the effects of temperature (temp), and acclimation (cooling time) as well as the interactive effects of the three factors. Statistically significant effects are indicated by bold F-values and p-values. Visualizations of the data can be seen in Figures 1.

|  | Agricultural soil | | | | | | Forest soil | | | | | |
| --- | --- | --- | --- | --- | --- | --- | --- | --- | --- | --- | --- | --- |
|  | temp | | cooling time | | temp x cooling time | | temp | | cooling time | | temp x cooling time | |
|  | F-value | p-value | F-value | p-value | F-value | p-value | F-value | p-value | F-value | p-value | F-value | p-value |
| MBC (mg C g^-1^ SOC) | 0.341 | 0.570 | **47.19** | **<0.001** | 1.828 | 0.201 | **6.154** | **0.035** | 4.539 | 0.062 | 0.599 | 0.459 |
| microbial respiration (µg C h^-1^ g^-1^ SOC) | **676.8** | **<0.001** | **56.66** | **<0.001** | **40.24** | **0.0001** | **785.7** | **<0.001** | **46.65** | **0.0001** | 1.676 | 0.228 |
| microbial growth (µg C h^-1^ g^-1^ SOC) | **74.64** | **<0.001** | **9.985** | **0.008** | 0.018 | 0.846 | **131.8** | **<0.001** | **6.222** | **0.030** | 0.012 | 0.916 |
| CUE | 0.203 | 0.663 | **12.69** | **0.006** | **20.54** | **0.001** | **5.484** | **0.047** | 0.060 | 0.813 | 0.006 | 0.806 |

Table S2 Table 2 Response ratios to cooling for the measured parameters and effects of the site (agricultural or forest, n=4), glucose addition (+C), and acclimation (cooling time) as well as the respective interactive effects on the RR. Statistically significant effects are indicated in bold F- values and p-values in the respective column.

|  |  |  | microbial respiration | microbial growth | CUE | label respiration | label in mic. biomass | label in PLFA |
| --- | --- | --- | --- | --- | --- | --- | --- | --- |
| agricultural |  | H2O 42 h | 3.30 ± 0.25 | 2.74 ± 0.35 | 0.89 ± 0.04 |  |  |  |
|  |  | +C 42 h | 2.32 ± 0.12 | 4.76 ± 0.40 | 1.72 ± 0.15 | 1.41 ± 0.05 | 0.78 ± 0.05 | 1.51 ± 0.20 |
|  |  | H2O 186 h | 2.61 ± 0.19 | 5.92 ± 0.94 | 1.72 ± 0.25 |  |  |  |
|  |  | +C 186 h | 1.75 ± 0.06 | 2.75 ± 0.41 | 1.43 ± 0.21 | 1.36 ± 0.02 | 0.97 ± 0.10 | 1.42 ± 0.20 |
| forest |  | H2O 42 h | 3.28 ± 0.04 | 4.26 ± 0.59 | 1.18 ± 0.11 |  |  |  |
|  |  | +C 42 h | 2.60 ± 0.13 | 8.56 ± 1.36 | 2.70 ± 0.53 | 1.20 ± 0.05 | 1.04 ± 0.14 | 1.6 ± 0.13 |
|  |  | H2O 186 h | 2.79 ± 0.12 | 3.43 ± 0.16 | 1.11 ± 0.05 |  |  |  |
|  |  | +C 186 h | 2.47 ± 0.09 | 2.59 ± 0.18 | 1.04 ± 0.07 | 1.34 ± 0.02 | 0.98 ± 0.14 | 1.19 ± 0.28 |
|  | Site | F-value | 3.199 | 1.536 | 0.006 | 5.659 | 1.010 | 0.026 |
|  |  | p-value | 0.124 | 0.228 | 0.941 | 0.055 | 0.335 | 0.875 |
|  | +C | F-value | **59.01** | 0.187 | **10.22** |  |  |  |
|  |  | p-value | **<0.001** | 0.670 | **0.004** |  |  |  |
|  | cooling time | F-value | **26.46** | **7.767** | 2.193 | 0.843 | 0.242 | 1.040 |
|  |  | p-value | **<0.001** | **0.011** | 0.152 | 0.394 | 0.632 | 0.330 |
|  | Site x +C | F-value | **5.188** | 2.051 | 0.544 |  |  |  |
|  |  | p-value | **0.035** | 0.166 | 0.468 |  |  |  |
|  | Site x cooling time | F-value | 2.948 | **14.28** | 15.58 | 4.109 | 0.911 | 0.494 |
|  |  | p-value | 0.103 | **0.001** | **<0.001** | 0.089 | 0.359 | 0.497 |
|  | +C x cooling time | F-value | 1.734 | **30.24** | 21.94 |  |  |  |
|  |  | p-value | 0.204 | **<0.001** | **<0.001** |  |  |  |
|  | Site x +C x cooling time | F-value | 0.445 | 0.614 | 0.009 |  |  |  |
|  |  | p-value | 0.513 | 0.441 | 0.925 |  |  |  |

Table S2 Statistical test results for agricultural soil (n=4). Shown are the effects of glucose addition (+C), temperature (temp), and acclimation (cooling time) as well as the interactive effects of the three factors. Statistically significant effects are indicated by bold F-values and p-values. Visualizations of the data can be seen in Figures 2, 4 and S1.

|  | +C | | temp | | Cooling time | | +C x temp | | +C x cooling time | | temp x cooling time | | +C x temp x cooling time | |
| --- | --- | --- | --- | --- | --- | --- | --- | --- | --- | --- | --- | --- | --- | --- |
|  | F-value | p-value | F-value | p-value | F-value | p-value | F-value | p-value | F-value | p-value | F-value | p-value | F-value | p-value |
| MBC (mg C g^-1^ SOC) | 0.433 | 0.5181 | **13.97** | **0.001** | **90.90** | **<0.001** | 0.992 | 0.331 | 2.556 | 0.126 | 0.944 | 0.343 | 1.530 | 0.230 |
| total PLFA content (µmol C g^-1^ SOC) | **11.77** | **0.002** | 0.326 | 0.577 | 0.001 | 0.974 | 0.002 | 0.961 | 0.004 | 0.948 | 0.005 | 0.943 | 0.216 | 0.647 |
| microbial respiration (µg C h^-1^ g^-1^ SOC) | 0.623 | 0.438 | **238.0** | **<0.001** | **9.126** | **0.006** | 0.666 | 0.423 | **21.02** | **0.0001** | 0.174 | 0.681 | **24.0** | **0.0001** |
| microbial growth (µg C h^-1^ g^-1^ SOC) | 2.090 | 0.162 | **141.4** | **<0.001** | **8.199** | **0.009** | 1.204 | 0.284 | 4.075 | 0.055 | 2.511 | 0.127 | 3.466 | 0.076 |
| CUE | **87.94** | **<.001** | **14.68** | **0.001** | 3.780 | 0.064 | **8.829** | **0.007** | **7.302** | **0.012** | **4.489** | **0.045** | **14.37** | **<.001** |
| label derived respiration (µg C h^-1^ g^-1^ SOC) |  |  | **33.15** | **0.001** | 2.972 | 0.113 |  |  |  |  | 0.307 | 0.591 |  |  |
| label incorporation in microbial biomass (µg C h^-1^ g^-1^ SOC) |  |  | 2.237 | 0.163 | **29.79** | **0.0002** |  |  |  |  | 0.014 | 0.907 |  |  |
| label incorporation in PLFA (µg C h^-1^ g^-1^ SOC) |  |  | **6.293** | **0.029** | 0.008 | 0.9283 |  |  |  |  | 0.0002 | 0.988 |  |  |
| label allocation to bacterial PLFAs (% of uptake) |  |  | 0.310 | 0.587 | 0.710 | 0.416 |  |  |  |  | 0.090 | 0.772 |  |  |
| label allocation to fungal PLFAs (% of uptake) |  |  | **12.36** | **0.008** | 3.414 | 0.102 |  |  |  |  | 0.355 | 0.568 |  |  |
| label allocation to unsaturated PLFAs (% of uptake) |  |  | **44.44** | **<.001** | 3.167 | 0.113 |  |  |  |  | 0.484 | 0.506 |  |  |

Table S3 Statistical test results for forest soil (n=4). Shown are the effects of glucose addition (+C), temperature (temp), and acclimation (cooling time) as well as the interactive effects of the three factors. Statistically significant effects are indicated by bold F-values and p-values. Visualizations of the data can be seen in Figures 2, 4 and S1.

|  | +C | | temp | | cooling time | | +C x temp | | +C x cooling time | | temp x cooling time | | +C x temp x cooling time | |
| --- | --- | --- | --- | --- | --- | --- | --- | --- | --- | --- | --- | --- | --- | --- |
|  | F-value | p-value | F-value | p-value | F-value | p-value | F-value | p-value | F-value | p-value | F-value | p-value | F-value | p-value |
| MBC C (g C g^-1^ SOC) | **8.893** | **0.007** | **5.480** | **0.028** | **19.09** | **0.0002** | 0.020 | 0.889 | **5.011** | **0.035** | 1.102 | 0.304 | 0.076 | 0.785 |
| total PLFA content (µmol C g^-1^ SOC) | 0.095 | 0.761 | 1.407 | 0.249 | **26.24** | **0.0001** | 0.658 | 0.427 | 1.457 | 0.242 | 0.0006 | 0.981 | 0.149 | 0.703 |
| microbial respiration (µg C h^-1^ g^-1^ SOC) | **120.5** | **<.0001** | **804.1** | **<.0001** | **40.79** | **<.0001** | **6.965** | **0.015** | 1.507 | 0.233 | **7.638** | **0.012** | 4.015 | 0.058 |
| microbial growth (µg C h^-1^ g^-1^ SOC) | **15.27** | **0.0006** | **349.2** | **<.0001** | 1.319 | 0.263 | 1.527 | 0.229 | **23.163** | **0.0001** | **10.58** | **0.004** | **9.223** | **0.006** |
| CUE | **24.64** | **<.001** | **25.17** | **<.001** | **12.27** | **0.002** | 0.995 | 0.330 | **12.74** | **0.002** | **7.147** | **0.015** | **9.509** | **0.006** |
| label derived respiration (µg C h^-1^ g^-1^ SOC) |  |  | **30.26** | **0.0004** | 3.338 | 0.101 |  |  |  |  | **8.868** | **0.016** |  |  |
| label incorporation in microbial biomass (µg C h^-1^ g^-1^ SOC) |  |  | 1.036 | 0.329 | 1.359 | 0.266 |  |  |  |  | 0.099 | 0.758 |  |  |
| label incorporation in PLFA (µg C h^-1^ g^-1^ SOC) |  |  | 2.026 | 0.192 | **6.680** | **0.032** |  |  |  |  | 1.591 | 0.243 |  |  |
| label allocation to bacterial PLFAs (% of uptake) |  |  | **16.15** | **0.002** | **7.298** | **0.021** |  |  |  |  | 2.048 | 0.180 |  |  |
| label allocation to fungal PLFAs (% of uptake) |  |  | 0.477 | 0.509 | **24.75** | **0.001** |  |  |  |  | **12.55** | **0.008** |  |  |
| label allocation to unsaturated PLFAs (% of uptake) |  |  | **73.56** | **<.001** | **11.47** | **0.006** |  |  |  |  | **10.72** | **0.007** |  |  |

Table S4 Results of Permutational Multivariate Analysis of Variance Using Distance Matrices to determine difference in PLFA-based microbial communities between sites as well as to determine effects of glucose addition (+C), temperature (temp), and acclimation (cooling time) as well as the interactive effects of the three factors. Statistically significant effects are indicated by bold R² and p-values. Visualizations of the data can be seen in Figure 3.

|  |  |  | agricultural soil | | forest soil | |
| --- | --- | --- | --- | --- | --- | --- |
|  | R² | p-value | R² | p-value | R² | p-value |
| Site | **0.752** | **0.001** |  |  |  |  |
|  |  |  |  |  |  |  |
| +C |  |  | **0.200** | **0.001** | 0.037 | 0.355 |
| temp |  |  | 0.010 | 0.851 | 0.027 | 0.531 |
| cooling time |  |  | **0.810** | **0.018** | 0.054 | 0.177 |
| +C x temp |  |  | 0.060 | 0.067 | 0.045 | 0.260 |
| +C x cooling time |  |  | 0.014 | 0.742 | 0.031 | 0.460 |
| temp x cooling time |  |  | 0.024 | 0.439 | 0.020 | 0.680 |
| +C x temp x cooling time |  |  | 0.608 | 0.979 | -0.009 | 1.000 |

|  | agricultural soil | | | | | | | |  | Forest soil | | | | | | | |
| --- | --- | --- | --- | --- | --- | --- | --- | --- | --- | --- | --- | --- | --- | --- | --- | --- | --- |
|  | 1°C +C 42 h | 1°C 42 h | 11°C +C 42 h | 11°C 42 h | 1°C +C 186 h | 1°C 186 h | 11°C +C 186 h | 11°C 186 h |  | 1°C +C 42 h | 1°C 42 h | 11°C +C 42 h | 11°C 42 h | 1°C +C 186 h | 1°C 186 h | 11°C +C 186 h | 11°C 186 h |
| Total soil C (mg C g^-1^ dry soil) | 9.748 ± 0.494 | 8.944 ± 0.143 | 9.248 ± 0.435 | 8.864 ± 0.185 | 6.408 ± 1.852 | 8.642 ± 0.138 | 8.777 ± 0.273 | 8.871 ± 0.186 |  | 40.53 ± 2.089 | 44.80 ± 4.204 | 45.21 ± 3.62 | 45.64 ± 2.048 | 40.540 ± 2.829 | 47.55 ± 2.84 | 38.97 ± 3.361 | 46.05 ± 2.984 |
| microbial biomass C (µg C g^-1^ dry soil) | 37.94 ± 2.715 | 38.28 ± 1.901 | 44.69 ± 0.491 | 45.15 ± 3.372 | 56.91 ± 2.956 | 60.60 ± 2.237 | 69.95 ± 6.491 | 59.17 ± 3.889 |  | 254.9 ± 16.95 | 263.9 ± 19.09 | 293.0 ± 21.40 | 294.3 ± 23.93 | 312.3 ± 25.47 | 301.0 ± 23.11 | 334.6 ± 22.07 | 333.1 ± 22.73 |
| total PLFA (nmol C g^-1^ dry soil) | 282.1 ± 6.146 | 180.1 ± 68.28 | 280.5 ± 29.54 | 145.3 ± 38.77 | 311.2 ± 33.21 | 162.8 ± 17.21 | 257.1 ± 56.62 | 158.5 ± 8.299 |  | 1873 ± 236.0 | 2533 ± 593.8 | 2010 ± 142.9 | 1975 ± 186.5 | 1414 ± 104.3 | 1175 ± 88.31 | 1325 ± 321.0 | 1048 ± 157.1 |
| bacterial PLFA (nmol C g^-1^ dry soil | 95.42 ± 2.043 | 68.56 ± 24.61 | 101.0 ± 11.39 | 52.90 ± 14.13 | 103.5 ± 12.89 | 58.13 ± 6.245 | 94.42 ± 21.31 | 56.80 ± 3.727 |  | 385.1 ± 46.31 | 538.1 ± 132.4 | 409.9 ± 28.49 | 415.7 ± 36.73 | 291.3 ± 28.67 | 241.6 ± 17.45 | 265.6 ± 63.86 | 216.5 ± 31.51 |
| fungal PLFA (nmol C g^-1^ dry soil | 38.05 ± 1.229 | 25.86 ± 9.517 | 39.61 ± 3.778 | 21.55 ± 5.476 | 45.50 ± 5.043 | 24.51 ± 2.449 | 36.39 ± 6.889 | 24.84 ± 1.148 |  | 399.2 ± 44.48 | 530.9 ± 98.11 | 437.6 ± 25.15 | 412.6 ± 25.80 | 290.1 ± 35.66 | 262.0 ± 26.91 | 278.4 ± 68.80 | 221.7 ± 28.30 |
| saturated PLFA (nmol C g^-1^ dry soil | 138.9 ± 2.520 | 85.11 ± 32.47 | 140.7 ± 15.01 | 68.62 ± 17.60 | 157.1 ± 15.90 | 77.69 ± 8.459 | 126.8 ± 29.41 | 73.51 ± 3.953 |  | 603.7 ± 69.54 | 815.0 ± 198.1 | 649.2 ± 46.45 | 655.6 ± 69.52 | 471.1 ± 36.26 | 371.7 ± 32.3 | 428.4 ± 105.5 | 327.8 ± 48.18 |
| unsaturated PLFA (nmol C g^-1^ dry soil | 143.2 ± 7.314 | 95.00 ± 35.83 | 139.8 ± 14.57 | 76.68 ± 21.20 | 154.1 ± 17.64 | 85.12 ± 8.840 | 130.3 ± 28.03 | 84.98 ± 4.539 |  | 1269.1 ± 167.8 | 1718 ± 395.8 | 1361 ± 97.31 | 1320 ± 117.7 | 943.1 ± 68.98 | 803.6 ± 56.27 | 896.5 ± 215.9 | 720.4 ± 109.5 |
| Fungi:bacteria | 0.400 ± 0.021 | 0.368 ± 0.011 | 0.396 ± 0.011 | 0.415 ± 0.011 | 0.443 ± 0.018 | 0.424 ± 0.020 | 0.401 ± 0.051 | 0.441 ± 0.023 |  | 1.041 ± 0.026 | 1.031 ± 0.049 | 1.076 ± 0.045 | 1.003 ± 0.032 | 0.992 ± 0.040 | 1.076 ± 0.031 | 1.040 ± 0.033 | 1.033 ± 0.035 |
| microbial respiration (ng C h^-1^ g^-1^ dry soil) | 69.37 ± 4.076 | 37.19 ± 2.837 | 159.5 ± 5.949 | 119.9 ± 2.149 | 71.49 ± 1.658 | 32.96 ± 2.303 | 124.8 ± 1.719 | 84.47 ± 2.955 |  | 448.9 ± 13.30 | 298.1 ± 20.99 | 1174 ± 85.78 | 975.2 ± 58.89 | 363.0 ± 18.48 | 257.2 ± 19.52 | 896.1 ± 48.90 | 712.4 ± 47.40 |
| microbial growth (ng C h^-1^ g^-1^ dry soil) | 16.30 ± 0.963 | 33.83 ± 4.982 | 76.41 ± 4.796 | 87.98 ± 7.694 | 22.58 ± 3.828 | 12.28 ± 1.199 | 55.82 ± 4.822 | 68.59 ± 6.824 |  | 65.51 ± 11.60 | 134.7 ± 3.698 | 499.4 ± 43.05 | 569.9 ± 75.08 | 162.9 ± 13.42 | 111.8 ± 14.36 | 415.9 ± 31.85 | 421.4 ± 23.46 |
| CUE | 0.191 ± 0.008 | 0.470 ± 0.017 | 0.324 ± 0.018 | 0.420 ± 0.023 | 0.236 ± 0.036 | 0.274 ± 0.029 | 0.307 ± 0.019 | 0.444 ± 0.02 |  | 0.124 ± 0.018 | 0.315 ± 0.021 | 0.298 ± 0.011 | 0.364 ± 0.023 | 0.309 ± 0.019 | 0.303 ± 0.034 | 0.316 ± 0.012 | 0.378 ± 0.014 |
| label derived respiration (ng C h^-1^ g^-1^ dry soil) | 16.42 ± 0.553 |  | 23.11 ± 0.670 |  | 17.27 ± 0.283 |  | 23.47 ± 0.265 |  |  | 83.35 ± 4.521 |  | 101.1 ± 10.04 |  | 67.00 ± 4.761 |  | 89.55 ± 4.698 |  |
| label in EOC (µg C g^-1^ dry soil) | 0.625 ± 0.088 |  | 0.265 ± 0.016 |  | 0.123 ± 0.027 |  | 0.489 ± 0.106 |  |  | 7.150 ± 0.875 |  | 5.041 ± 0.722 |  | 0.345 ± 0.038 |  | 7.116 ± 1.617 |  |
| label incorporation in microbial biomass (ng C h^-1^ g^-1^ dry soil) | 75.57 ± 4.939 |  | 58.29 ± 1.274 |  | 89.89 ± 7.486 |  | 84.56 ± 2.358 |  |  | 222.6 ± 9.137 |  | 229.4 ± 28.91 |  | 255.1 ± 28.03 |  | 248.7 ± 40.79 |  |
| label incorporation in PLFA (ng C h^-1^ g^-1^ dry soil) | 28.04 ± 1.318 |  | 41.52 ± 4.677 |  | 28.84 ± 4.839 |  | 40.84 ± 9.231 |  |  | 95.57 ± 13.93 |  | 149.7 ± 17.78 |  | 85.66 ± 30.13 |  | 78.71 ± 29.61 |  |
| label allocation to bacterial PLFAs (% of uptake) | 29.42 ± 0.541 |  | 29.58 ± 0.282 |  | 28.33 ± 1.135 |  | 29.04 ± 0.915 |  |  | 23.91 ± 0.091 |  | 20.41 ± 0.678 |  | 24.64 ± 0.898 |  | 22.96 ± 0.323 |  |
| label allocation to fungal PLFAs (% of uptake) | 27.69 ± 1.005 |  | 26.84 ± 0.574 |  | 29.83 ± 1.036 |  | 27.87 ± 1.702 |  |  | 18.67 ± 0.93 |  | 22.67 ± 2.317 |  | 16.83 ± 2.034 |  | 15.14 ± 1.061 |  |
| label allocation to saturated PLFAs (% of uptake) | 28.13 ± 0.782 |  | 32.72 ± 0.753 |  | 30.37 ± 0.92 |  | 33.95 ± 1.997 |  |  | 24.35 ± 0.626 |  | 28.43 ± 0.816 |  | 24.23 ± 0.446 |  | 33.38 ± 0.594 |  |
| label allocation to unsaturated PLFAs (% of uptake) | 71.87 ± 0.782 |  | 67.28 ± 0.753 |  | 69.63 ± 0.92 |  | 66.05 ± 1.997 |  |  | 75.65 ± 0.626 |  | 71.57 ± 0.816 |  | 75.77 ± 0.446 |  | 66.62 ± 0.594 |  |

Table S4: Mean values and standard errors of measured parameters.


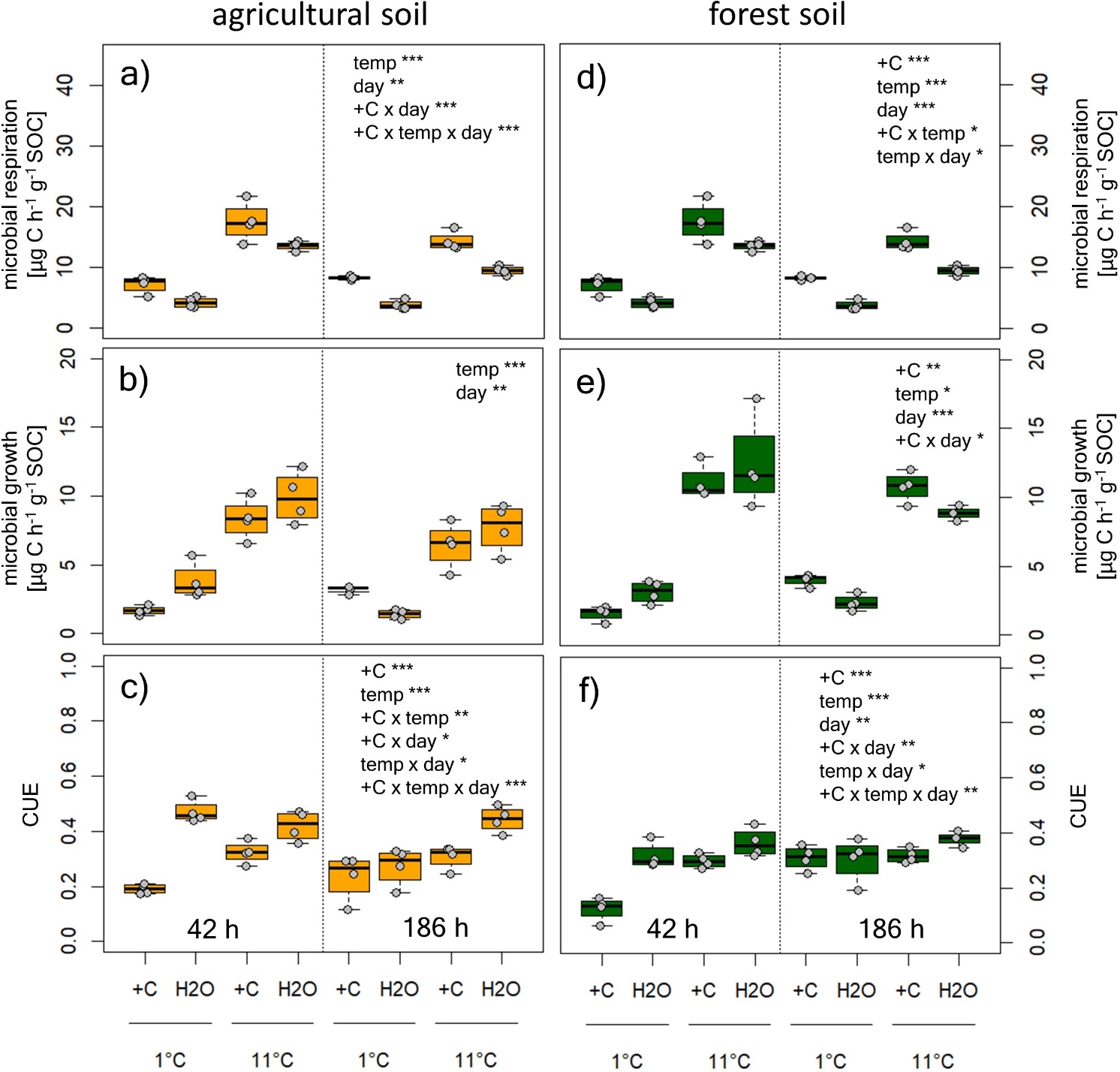


Figure S1: Microbial Respiration, growth (both calculated per g SOC), and carbon use efficiency of agricultural soil (a-c) and forest soil (d-f) in response to glucose (+C) or water addition (H2O) at different temperatures (1 °C and 11 °C) during immediate cooling (42 h) and after acclimation to the temperatures (186 h). Statistics accompanying this figure can be found in Table S2 and Table S3.
